# Complete mitochondrial genome of gray black carp (*Mylopharyngodon piceus*)

**DOI:** 10.1080/23802359.2020.1765206

**Published:** 2020-05-13

**Authors:** Sheng-cheng Bao, Nan Xie, Xiao-yan Xu, Yu-hong Su, Tian-jie Bao, Yu-bang Shen, Jia-le Li

**Affiliations:** a Key Laboratory of Freshwater Aquatic Genetic Resources, Ministry of Agriculture, Shanghai Ocean University, Shanghai, China; b Hangzhou Academy of Agricultural Sciences, Hangzhou, China; c Shanghai Engineering Research Center of Aquaculture, Shanghai Ocean University, Shanghai, China; d National Demonstration Center for Experimental Fisheries Science Education, Shanghai Ocean University, Shanghai, China

**Keywords:** *Mylopharyngodon piceus*, gray black carp, mitochondrial genome, phylogenetic analysis

## Abstract

The black carp (*Mylopharyngodon piceus*), native to eastern Asian, is a large, commercially valuable fish, and has been widely introduced to other countries. In this study, the mitochondrial gene sequence of gray black carp (*M. piceus* MT084757) in Foshan, Guangdong Province was first determined using the Sanger sequencing method. The mitochondrial DNA genome was 16,616 bp in length, including 13 protein-coding genes (PCGs), two rRNA genes, 22 transfer RNA genes, and a non-coding control region (D-loop). The overall nucleotide composition of the mitochondrial DNA is 32.04% A, 24.52% T, 15.68% C, 27.76% G, with 56.56% AT, respectively. Phylogenetic tree analysis suggests that the gray black carp (*M. piceus* MT 084757) is closely related to *Elopichthys bambus* and *Squaliobarbus curriculus*. The complete mitochondrial genome of the gray black carp (*M. piceus* MT 084757) would be useful for researching the causes of changes in body color.

The black carp (*Mylopharyngodon piceus*) is an important economic and ecological freshwater fish with a wide distribution range from the Pearl River to the Amur River, mainly in the plains south of the Yangtze River in China (Chen 1998; Xue-Ping 2016). Black carp (*M. piceus*), grass carp (*Ctenopharyngodon idella*), silver carp (*Hypophthalmichthys molitrix*), and bighead carp (*Hypophthalmichthys nobilis*) have contributed a lot to China’s aquaculture industry, so they are called “four famous farm fishes” in China (Li 1998). Black carp feeds on mollusks and are often used to control the overproduction of these organisms in freshwater aquaculture facilities (Ben-Ami and Heller 2001). It has been introduced to North America, Europe, Africa, and the Middle East (Haag 2008).

The gray black carp (*M. piceus* MT084757) was collected from Foshan, Guangdong (23°02’N, 113°06’E) in China, and the complete mitochondrial gene sequence was determined using the Sanger sequencing method. All samples (SHOUBC72) have been deposited in the College of Fisheries and Life Science, Shanghai Ocean University, Shanghai, China. Total genomic DNA was extracted from a single specimen (SHOUBC72_1) using the improved method with multi-well plates (Pall Corporation) (Yue and Orban 2005). Subsequently, based on the existing complete mitochondrial gene of the black carp (*M. piceus* NC_011141.1) (Wang et al. 2012), 29 pairs of primers were designed, the samples were amplified by PCR, and then sequenced using Sanger sequencing technology. The mitogenome was assembled using *M. piceus* NC_011141.1 as reference. The identification and location of protein-coding genes were determined by *M. piceus* NC_011141.1 too. The program tRNAscan-SE-2.0.5.tar.gz was used to predict the tRNA genes (http://lowelab.ucsc.edu/tRNAscan-SE/) (Chan and Lowe 2019). A physical map of the genome was generated using GenomeVX (Conant and Wolfe 2008). The analysis of other nucleotide/amino acid components and the construction of the evolutionary tree were using MEGA7 (Kumar et al. 2016).

The complete genome sequence of the gray black carp (*M. piceus* MT 084757) is 16,616 bp in size. The overall base composition was 32.04%A, 24.52%T, 15.68%C, 27.76%G, with 56.56% AT, respectively, containing 13 protein coding-genes (PCGs), two rRNA genes, 22 transfer RNAs genes, and a non-coding control region (D-loop). 11 PCGs started with ATG, but CO1 uses GTG and ND5 uses TTG as the start codon; Five PCGs were finished with TGA. The incomplete stop codon (TA–, T––) were found in seven genes (ND1, ND2, ND4L, ND5, ND6, ATP6, CO1, and CYTB). Gene overlap was observed at the border of eight genes (tRNA^Ile^-tRNA^Gln^, ATP6-ATP8, ND4-ND4L, and tRNA^Thr^-tRNA^Pro^). All 22 tRNAs distributed on the H and L strands were between 68 and 76 bp in length. 14 tRNA genes were encoded on the H and eight on the L strands. Most of tRNAs could form a common cloverleaf secondary structure, except tRNA^Ser(AGY)^ gene without DHU stem (Sprinzl 1998; Chan and Lowe 2019). Two rRNA genes, 12SRNA, and 16SRNA were 964 bp and 1692 bp in size, respectively, which located between the tRNA^Phe^ and tRNA^Leu(UUR)^ and separated by the tRNA^Val^ gene. The control region was located between tRNA^Phe^ and tRNA^Pro^.

Based on the complete sequences of the mitochondrial genomes of 14 species, a phylogenetic tree was constructed using the neighbour-joining method (Figure 1). The phylogenetic tree suggested that the gray black carp (*M. piceus* MT 084757) is closely related to *Elopichthys bambus* and *Squaliobarbus curriculus.*

**Figure 1.**
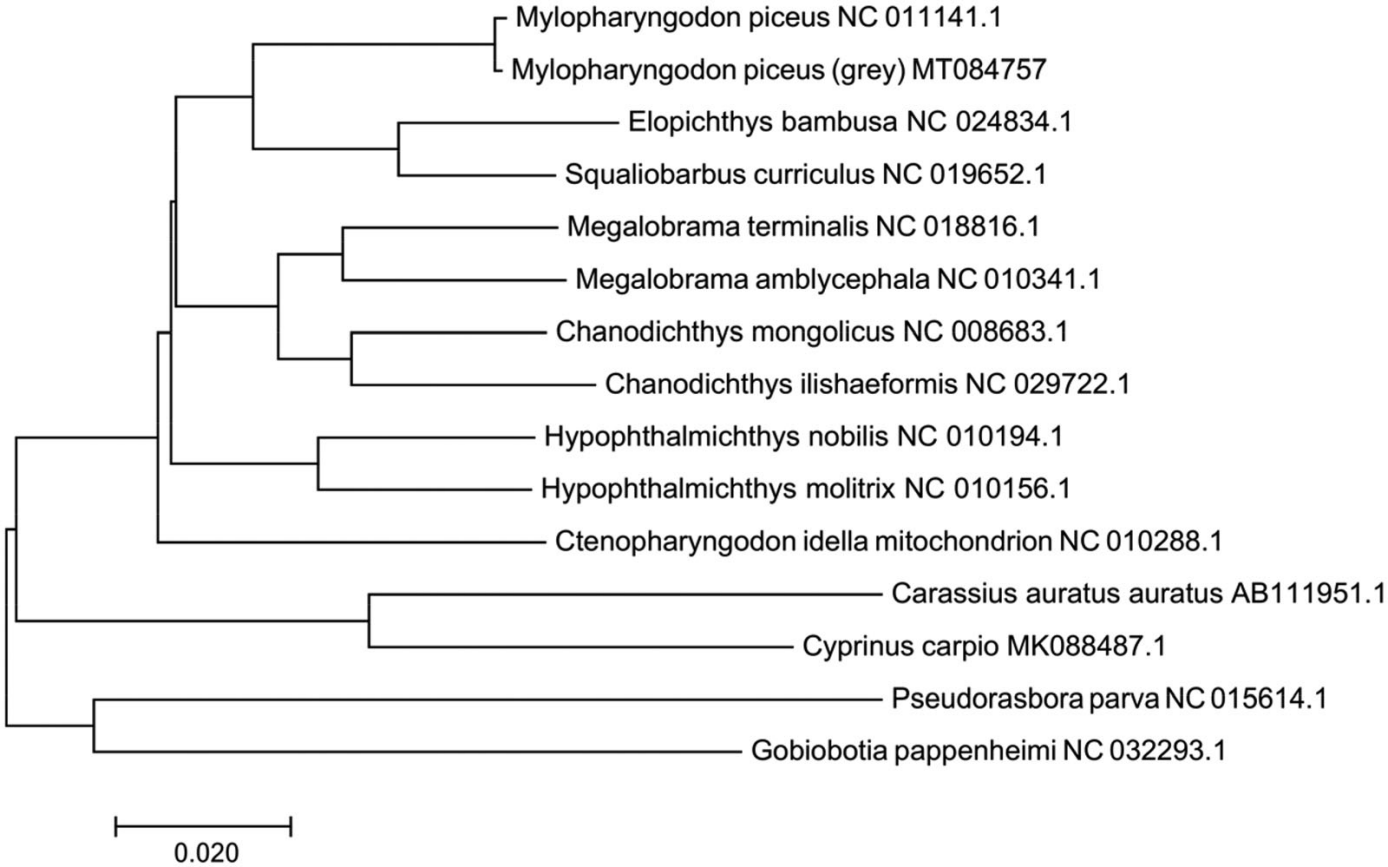
Construction of neibour-joining (NJ) phylogenetic tree was based on complete mitochondrial sequences of 14 species. GenBank accession numbers are given after the species name.

## Data Availability

The data that support the findings of this study are openly available in NCBI at https://www.ncbi.nlm.nih.gov/nuccore/MT084757, reference numberMT084757.
